# Recurrent Hydropneumothorax as an Initial Presentation of Malignant Pleural Mesothelioma: Diagnostic Challenges and the Evolving Role of Pleural Fluid Cytology

**DOI:** 10.7759/cureus.114913

**Published:** 2026-08-21

**Authors:** Astrid Goedseels, Els De Droogh, Paul Hollering, Marjan Hertoghs, Elise Peys

**Affiliations:** 1 Pulmonology, Ziekenhuis aan de Stroom, Antwerp, BEL; 2 Thoracic Surgery, Ziekenhuis aan de Stroom, Antwerp, BEL; 3 Pathology, Ziekenhuis aan de Stroom, Antwerp, BEL

**Keywords:** bap-1, malignant pleural mesothelioma (mpm), pleural malignancy, recurrent hydropneumothorax, vats, video-assisted thoracoscopic surgery

## Abstract

Malignant pleural mesothelioma (MPM) is an aggressive pleural malignancy typically associated with asbestos exposure and characterized by a prolonged latency period, often resulting in delayed diagnosis. Although pleural effusion and pleural thickening are common presentations, recurrent hydropneumothorax is an uncommon initial manifestation. Given the broad differential diagnosis of hydropneumothorax, recurrent cases require a thorough clinical and occupational history and appropriate diagnostic evaluation to avoid overlooking an underlying MPM. This case report describes an uncommon initial presentation of MPM and discusses when it is necessary to obtain a tissue sample to confirm the diagnosis.

## Introduction

Malignant pleural mesothelioma (MPM) is a rare aggressive pleural malignancy. In Belgium, the incidence has plateaued at approximately 300 cases per year since 2015, corresponding to an incidence of approximately 2.7 cases per 100,000 inhabitants in a population of 10-11 million [1]. It is almost invariably associated with previous asbestos exposure and characterized by a long latency period between asbestos exposure and clinical manifestation, often resulting in a late presentation. Symptoms are typically nonspecific, ranging from exertional dyspnea to chest pain, occasionally accompanied by cough and weight loss. A spontaneous hydropneumothorax represents an uncommon initial presentation of MPM. Early diagnosis remains challenging because imaging findings may initially be absent. Cytological examination of pleural effusion is generally considered insufficient for diagnostic confirmation, with a sensitivity of 20-30% [2]. However, tissue biopsy remains the recommended diagnostic standard [3]. Here we report the case of a 72-year-old man presenting with a recurrent left-sided hydropneumothorax without known asbestos exposure.

## Case presentation

A 72-year-old man presented to the emergency department with exertional dyspnea and mild left-sided thoracic pain, both progressively worsened over the preceding month. He denied fever; he was a never-smoker, and his past medical history was unremarkable for lung diseases. On presentation, he was hemodynamically stable with moderate hypoxemia necessitating 2 L of supplemental oxygen.

**Figure 1:**
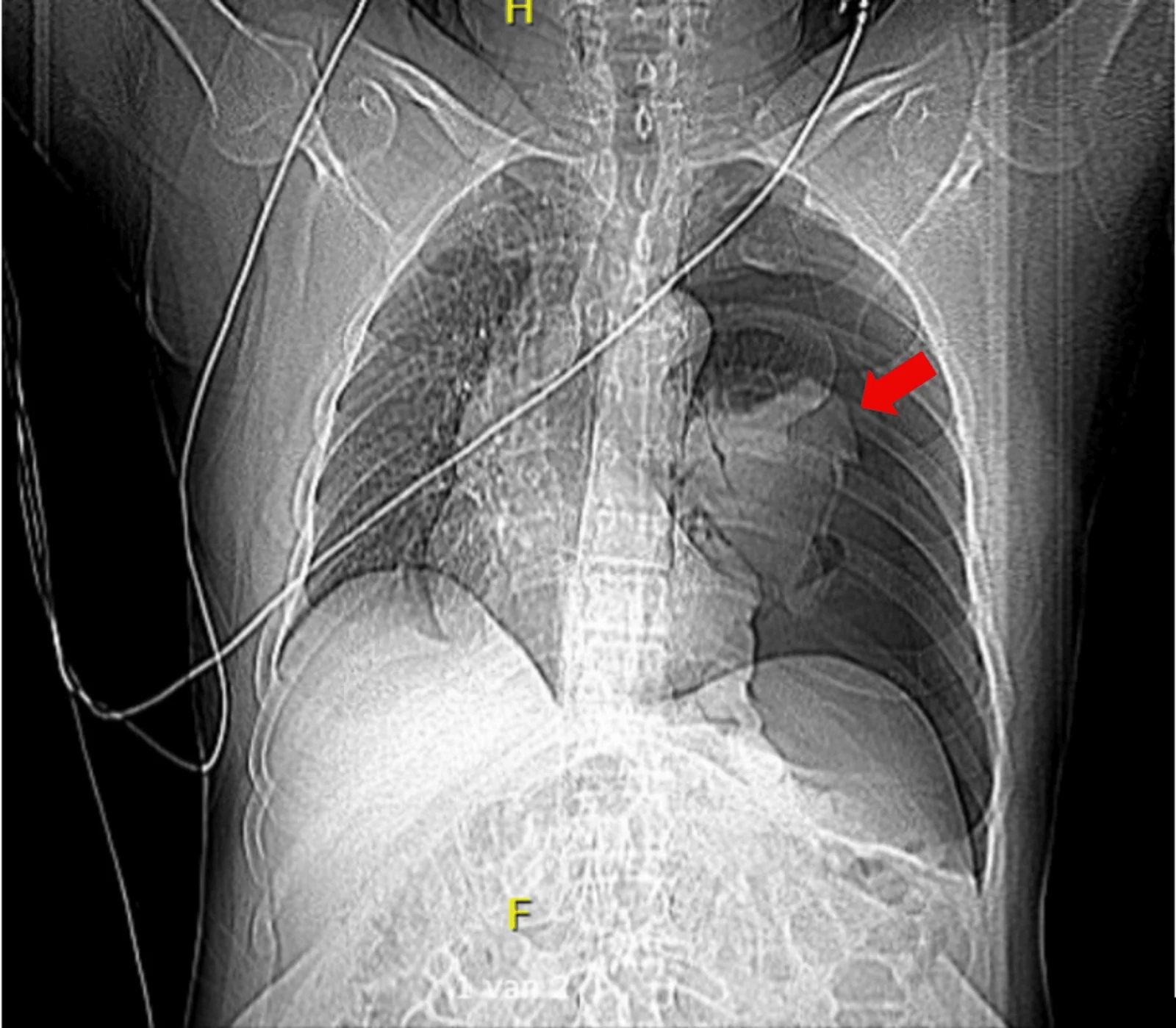
Initial CT scan demonstrating left-sided hydropneumothorax with collapse of the left lung. The red arrow indicates the collapsed left lung. CT: computed tomography

**Figure 2:**
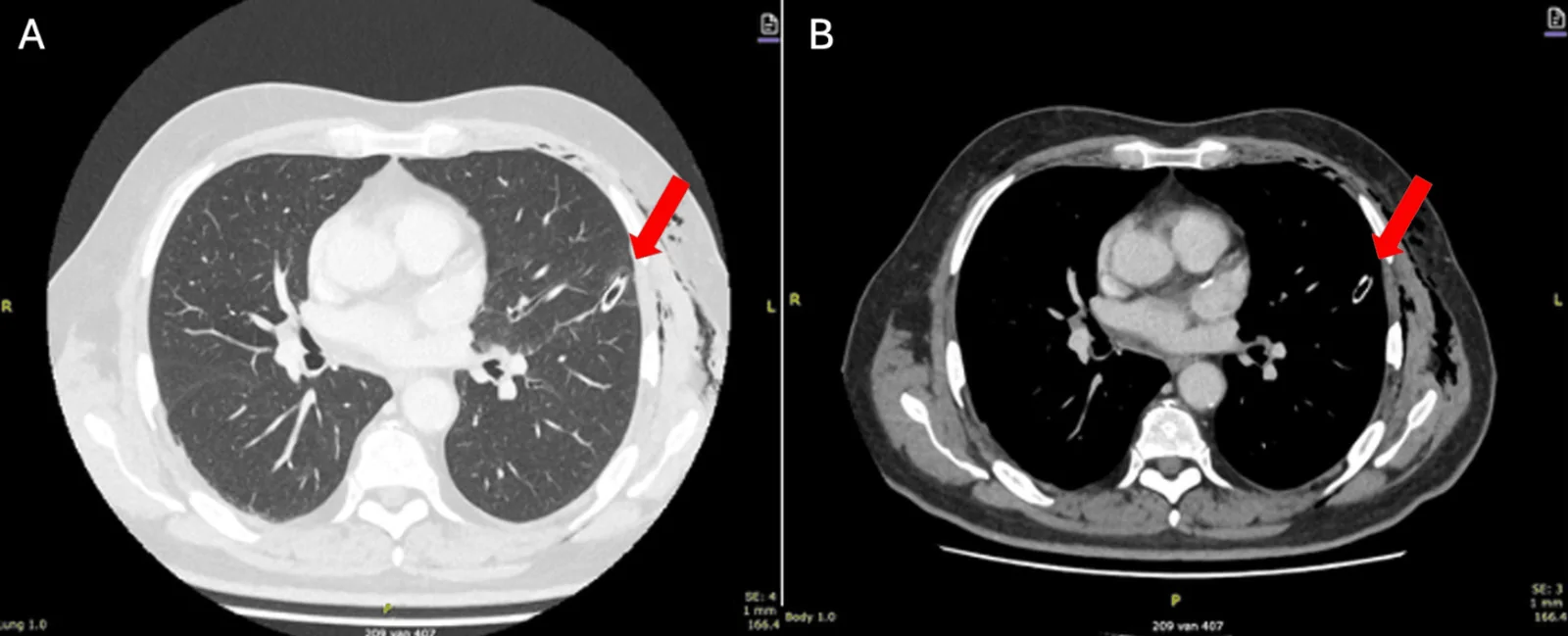
CT imaging with chest drain in situ and no signs of pleural abnormalities. (A) Lung-window images with the chest drain in situ, indicated by the red arrow. (B) Mediastinal-window images with the chest drain in situ, indicated by the red arrow. CT: computed tomography

**Figure 3:**
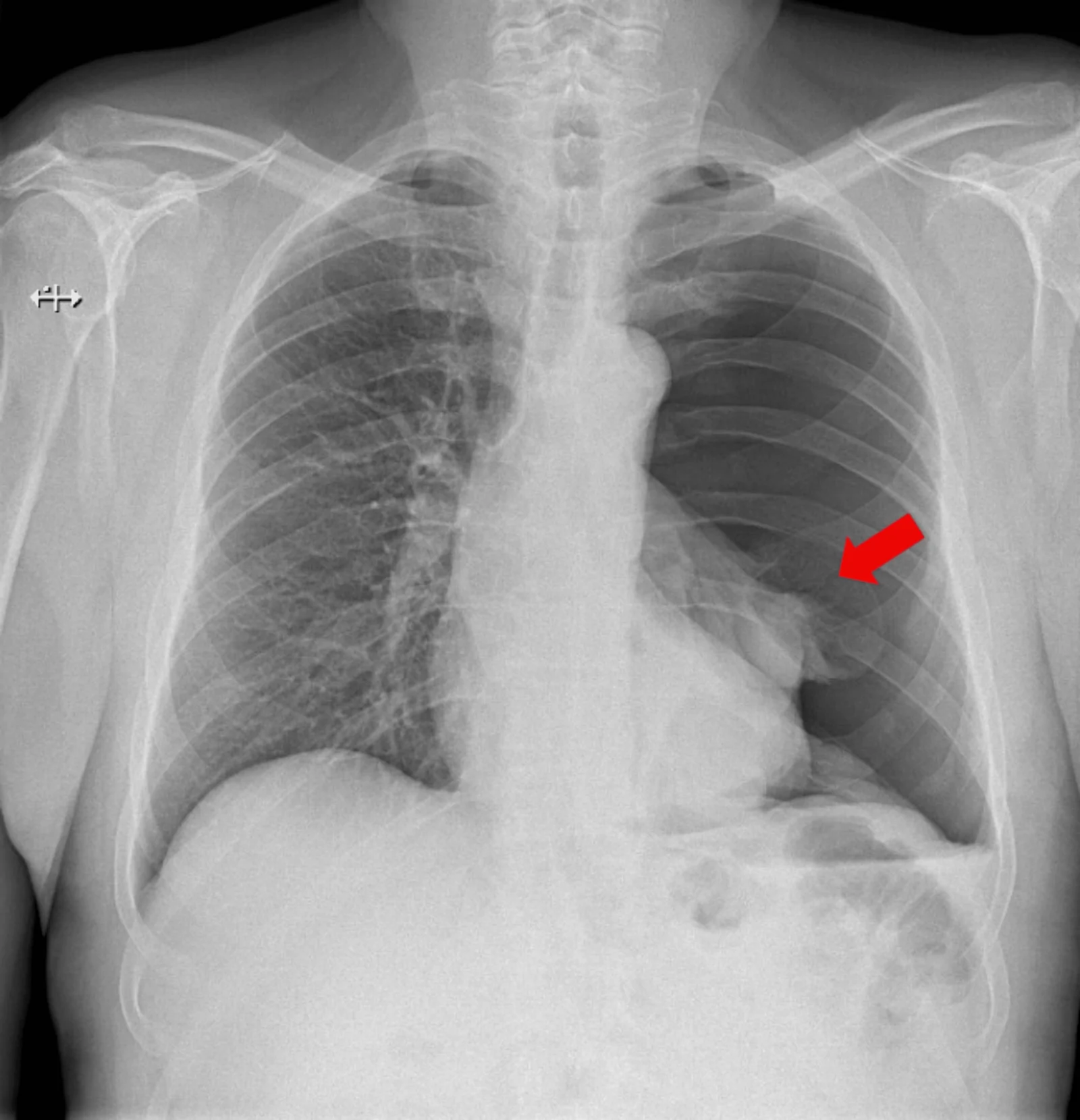
Chest radiograph obtained one week later demonstrating recurrent collapse of the left lung. The red arrow indicates the recurrently collapsed left lung.

Initial diagnostic work-up revealed a collapsed lung on the left side with a small associated pleural effusion (Figure 1). A chest drain was inserted under local anesthesia, evacuating a small amount of pleural fluid. The fluid was sent for microbiological cultures and cytological and biochemical analyses. The patient was admitted to the pulmonary department with the thoracic drain on suction. Over the next few days, the lung re-expanded, and a follow-up computed tomography (CT) was performed to investigate the cause of a spontaneous hydropneumothorax in a never-smoker. The CT scan showed no evidence of malignancy or bullae (Figure 2). The chest drain was removed without recurrence of pneumothorax; the patient felt better, and he was discharged. During the outpatient follow-up visit one week later, a chest radiograph revealed a recurrent collapse of the left lung (Figure 3). Clinically, the patient reported symptomatic improvement compared to his initial presentation. He remained hemodynamically stable and did not require supplemental oxygen.

**Figure 4:**
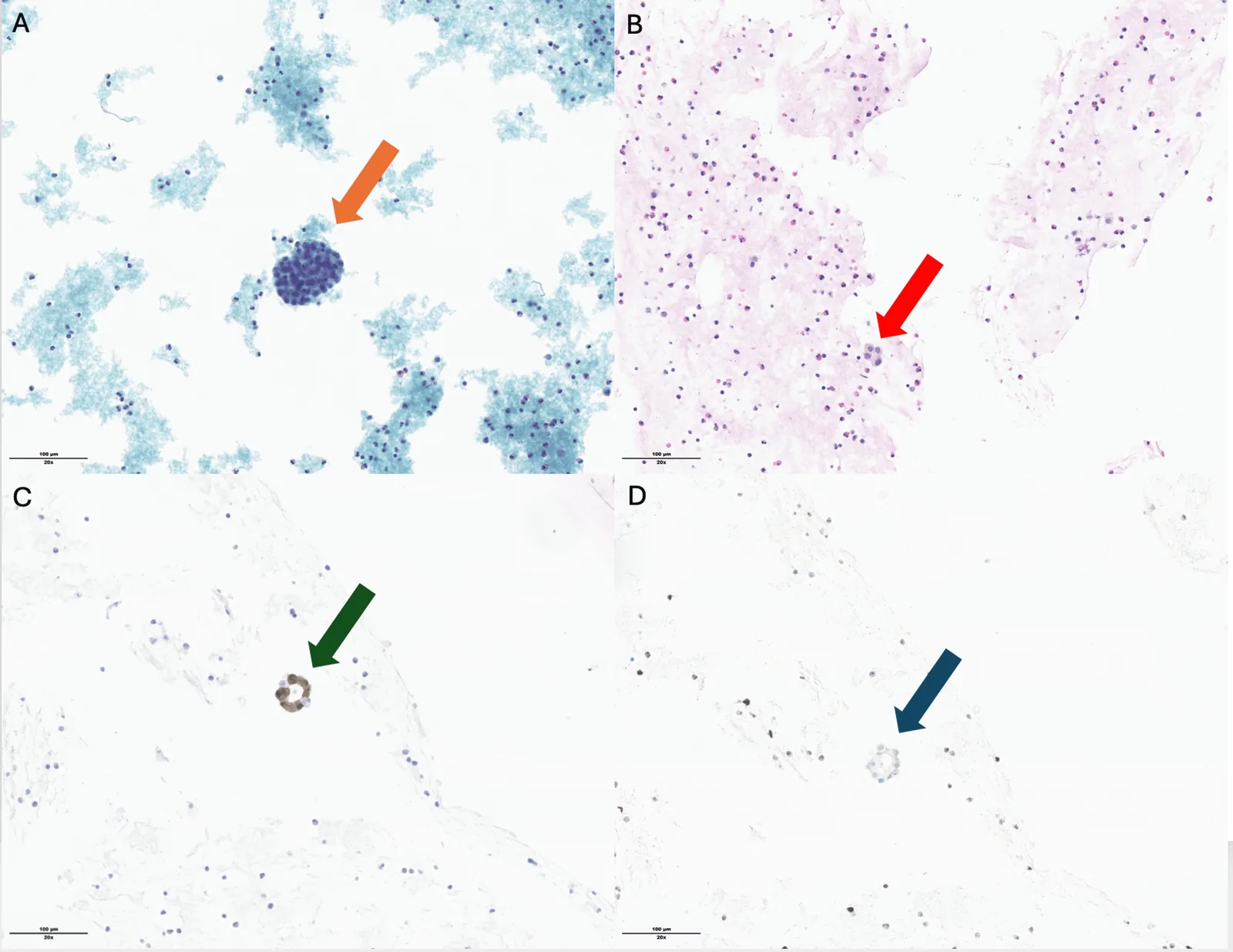
Histopathology of the pleural fluid. (A) Thin-layer Papanicolaou-stained cytological preparation of the pleural fluid, showing a group of atypical mesothelial cells, indicated by the orange arrow (20x magnification, scale bar 100 μm). (B) Pleural fluid hematoxylin and eosin staining demonstrating a small group of atypical mesothelial cells within an inflammatory background, indicated by the red arrow (20x magnification, scale bar 100 μm). (C) Pleural fluid cell block with calretinin immunohistochemical staining, confirming the mesothelial origin of the atypical cells, indicated by the green arrow (20x magnification, scale bar 100 μm). (D) Pleural fluid cell block with BAP1 immunohistochemical staining, demonstrating the loss of nuclear BAP1 expression in the mesothelial cells, indicated by the blue arrow (20x magnification, scale bar 100 μm).

By the time of the outpatient visit, pleural fluid results had become available. They revealed eosinophilic inflammation and a small cluster of atypical mesothelial cells with loss of BRCA1-associated protein 1 (BAP-1) expression, and with immunohistochemical markers positive for calretinin, cytokeratin 5 (CK5), and Wilms’ tumor 1 (WT1) (Figure 4). As loss of BAP-1 expression is highly specific for malignant mesothelial proliferation, these findings established the presence of a malignant mesothelial process [4]. Nevertheless, thoracoscopic pleural biopsy was pursued because MPM had not been clinically suspected in the absence of known asbestos exposure; the cytological specimen was sparsely cellular and the inflammatory background raised concern regarding the representativeness of the sample. Histological confirmation was therefore obtained. Other infectious and inflammatory etiologies had been excluded.

**Figure 5:**
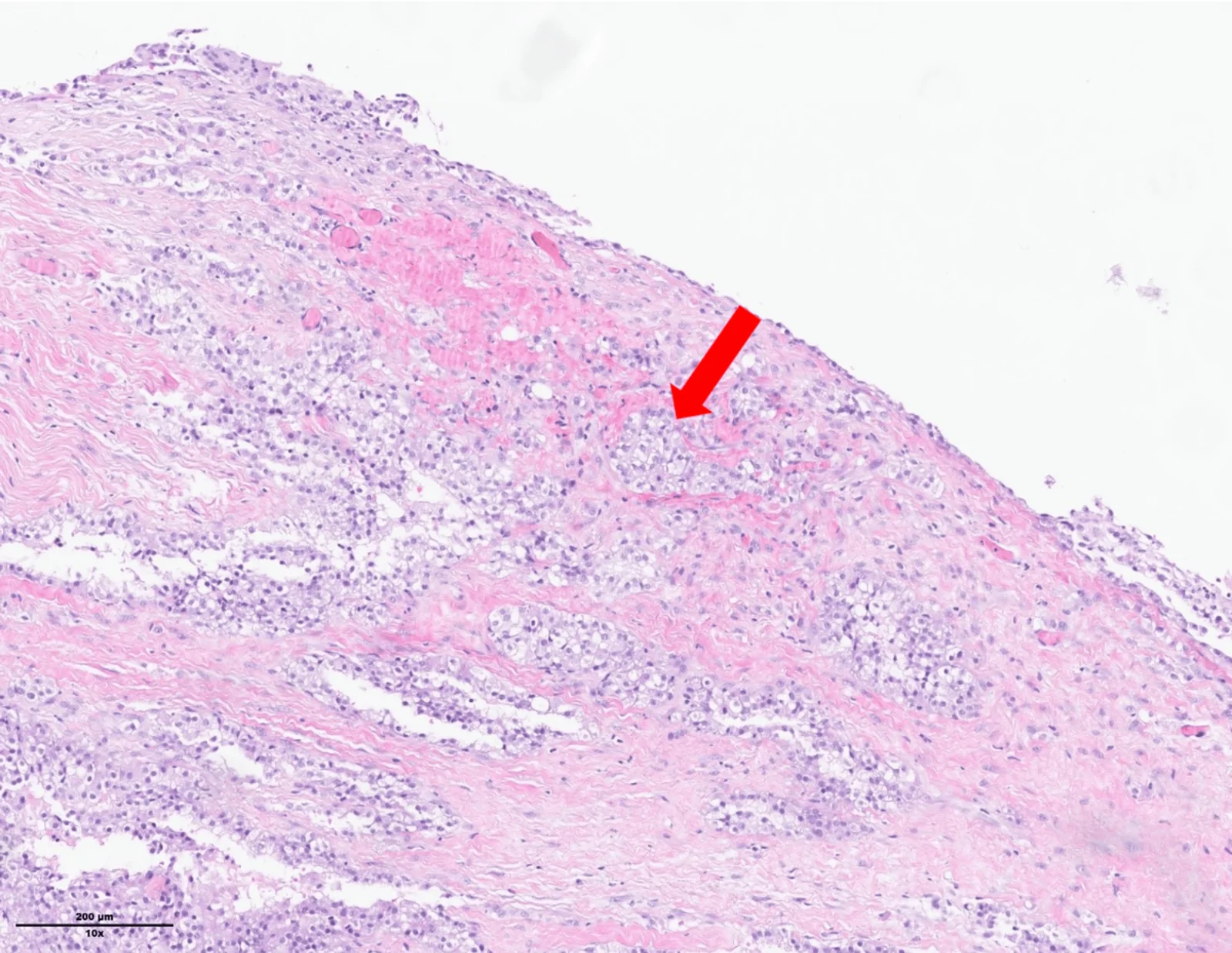
Pleural biopsy with hematoxylin and eosin staining, demonstrating an infiltrative proliferation of atypical epithelioid cells within fibrous pleural tissue, extending into the underlying adipose tissue, indicated by the red arrow (10x magnification, scale bar 200 μm).

**Figure 6:**
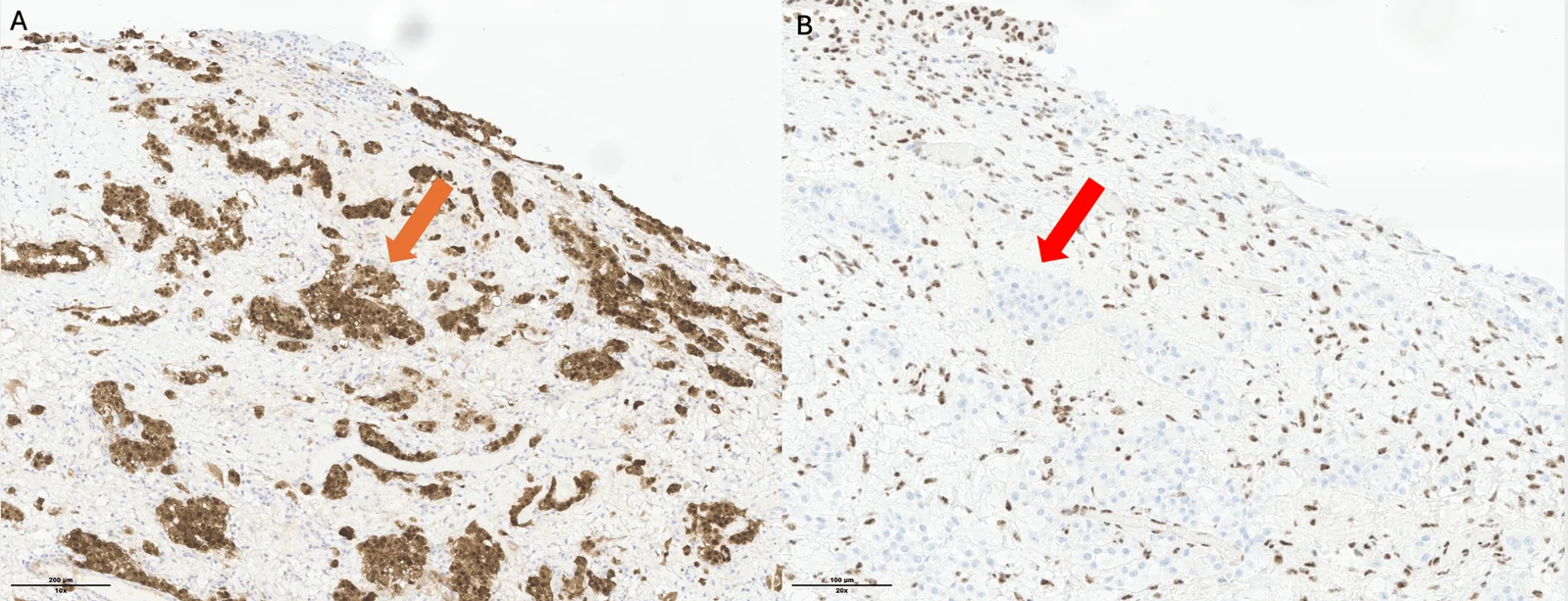
Histopathology of the pleura. (A) Pleural cell block with calretinin immunohistochemical staining, confirming the mesothelial origin of the atypical cells, indicated by the orange arrow (10x magnification, scale bar 200 μm). (B) Pleural cell block with BAP1 immunohistochemical staining, demonstrating the loss of nuclear BAP1 expression in the mesothelial cells, indicated by the red arrow (20x magnification, scale bar 100 μm).

The patient underwent a left video-assisted thoracoscopic surgery (VATS) with pleural biopsies, apical pleurectomy, and bullectomy (as there was a small apical bulla seen at surgery). No talc pleurodesis was performed. A new chest drain was placed, which was removed after a few days following full lung re-expansion. Macroscopic evaluation of the lung biopsy from the apical bleb showed normal lung parenchyma. Microscopic evaluation showed several foci of atypical cells along the surface of the visceral pleura without evidence of infiltration into the visceral pleura or the underlying lung parenchyma. Immunohistochemical staining was positive for WT1 and demonstrated loss of BAP-1 expression, an immunohistochemical finding highly supportive of malignant mesothelial proliferation. Macroscopic evaluation of the pleural biopsies revealed a smooth pleural surface. Microscopic evaluation showed an infiltrative population of atypical cells within the pleura extending into the adipose tissue (Figure 5). Immunohistochemical staining was positive for calretinin, CK5, epithelial membrane antigen (EMA), and WIT1, and again showed loss of BAP-1 expression (Figure 6). Based on the histopathological findings, the diagnosis of epithelioid mesothelioma was confirmed.

A subsequent positron emission tomography-CT (PET-CT) showed no fluorodeoxyglucose (FDG) uptake. The case was reviewed by the Belgian mesothelioma board, which confirmed the diagnosis of MPM.

The patient was offered participation in an ongoing study to re-evaluate operability after two cycles of chemo-immunotherapy. However, he considered this approach too invasive and was therefore closely monitored with a follow-up CT scan after three months. 

## Discussion

In this case, we describe the diagnosis of MPM in a never-smoker without known asbestos exposure, presenting with the known, yet rare, initial manifestation of a hydropneumothorax [5]. Although asbestos exposure is the main risk factor, approximately 20% of MPM occur without a known history of asbestos exposure [6]. The typical clinical presentation of MPM includes dyspnea, cough, and chest discomfort, with imaging often demonstrating pleural thickening or plaques, with or without chest wall invasion, and occasionally accompanied by pleural effusion.

The diagnostic challenge of MPM lies in its nonspecific symptomatology and the possible absence of a clear history of asbestos exposure. Although thoracoscopic pleural biopsy has traditionally been regarded as the diagnostic reference standard, loss of BAP-1 expression has substantially improved the diagnostic value of pleural fluid cytology. As BAP-1 loss is highly specific for malignant mesothelial proliferation, tissue biopsy is not invariably required to establish malignancy. However, biopsy remains important for clinicopathological correlation and histological subtyping, particularly in cases with limited cellularity or an unexpected clinical presentation. VATS pleural biopsy retains an excellent diagnostic yield of approximately 95% [7]. In this case, the patient initially presented with only nonspecific symptoms, including progressive dyspnea and chest discomfort, and the initial CT scan did not reveal any pleural abnormalities. Furthermore, a recent study demonstrated that CT scan has a negative predictive value of around 65% for the diagnosis of pleural malignancy. This finding emphasizes the importance of invasive diagnostic procedures in patients with a suspicious or otherwise unexplained clinical presentation [8].

Only a few case reports have described recurrent hydropneumothorax as the early indicator of MPM [5,9-11]. The proposed pathogenesis of pneumothorax in this context includes rupture of a necrotic tumor mass. In our patient, no large cystic changes of bulky neoplastic lesions were present. However, there was tumor extension along the pleural surface with invasion into the adipose tissue. This relatively early stage of MPM may lead to pneumothorax in the absence of direct invasion into the lung parenchyma, likely due to disruption of the visceral pleura, as documented in two similar case reports [9,11]. This case illustrates that recurrent spontaneous hydropneumothorax should prompt consideration of MPM, even in patients without known asbestos exposure and with initially normal imaging. 

## Conclusions

MPM should be considered in patients presenting with recurrent spontaneous hydropneumothorax, even in the absence of known asbestos exposure or radiological evidence of pleural disease. Loss of BAP-1 expression in pleural fluid cytology can provide highly specific evidence of malignant mesothelial proliferation. Although tissue biopsy is not invariably required to establish malignancy, it remains valuable for clinicopathological correlation when cytological findings are limited, or the clinical suspicion is low. This case highlights the importance of early advanced diagnostic evaluation when hydropneumothorax recurs, even in the absence of abnormalities on CT or PET/CT imaging.
